# Chromophobe renal cell carcinoma – a rare kidney cancer with limited therapy options: a narrative review

**DOI:** 10.1186/s12894-025-01816-5

**Published:** 2025-06-02

**Authors:** Milad Pashai Fakhri, Jürgen Serth, Jan Hinrich Bräsen, Philipp Ivanyi, Markus Antonius Kuczyk, Hossein Tezval

**Affiliations:** 1 Department of Urology and Urological Oncology, Hannover Medical School, Hannover, 30625 Germany; 2 Nephropathology Unit, Institute of Pathology, Hannover Medical School, Hannover, Germany; 3 Department of Hematology, Hemostasis, Oncology and Stem Cell Transplantation, Hannover Medical School, Hannover, Germany

**Keywords:** Chromophobe renal cell carcinoma, Progesterone receptor, Sarcomatoid dedifferentiation, Genetics, Epigenetics, Checkpoint inhibitors, Grading system, Prognosis

## Abstract

Chromophobe renal cell carcinoma (chRCC) is a rare subtype of renal cell carcinoma (RCC) and is the most common form of non-clear cell renal cell carcinoma in young women. Compared to clear cell renal cell carcinoma (ccRCC), chRCC usually has an excellent prognosis, indicating the need for a reliable differential diagnosis, especially to distinguish it from eosinophilic variants of ccRCC. Another important differential diagnosis is renal oncocytoma (RO), which remains a major challenge even for experienced pathologists. The treatment of RO typically involves active surveillance, with surgical resection indicated if there is significant tumor growth. In contrast, for chRCC, the approach depends on tumor size, with either partial or radical nephrectomy being required. This review therefore summarizes key unique features and recent findings on this tumor, aiming to ensure a reliable differential diagnosis, thereby facilitating appropriate treatment selection and prognosis assessment. The histology of chRCC, including both for the classic and the eosinophilic subtype, is characterized by the appearance of raisinoid cell nuclei with perinuclear halos on microscopic imaging. In rare cases, signs of sarcomatoid, glandular and/or anaplastic dedifferentiation can also be observed, which significantly worsens the prognosis. The immunohistochemical marker phospho-S6 can be used to detect these changes. In addition to other routinely used markers such as C-Kit, CK7, EpCAM, CAIX and Claudin 7, we recommend the use of progesterone receptors as markers, as many chRCC express them and are thus progesterone-sensitive. This progesterone sensitivity could indicate that chRCC, similar to breast cancer, may represent a contraindication for the use of hormonal contraceptives. In addition to immunohistochemistry, molecular features of chRCC such as genetic, epigenetic, transcriptomic and proteomic alterations can be considered in the differential diagnosis. In this review, we therefore outline the most important established alterations in this context. In the treatment of metastatic chRCC, checkpoint inhibitors and tyrosine kinase inhibitors have demonstrated efficacy and may represent a promising new approach for managing dedifferentiated, aggressive or metastatic chRCC. This review aims to present recent therapeutic advances and provide innovative approaches for future clinical treatment decisions.

## Background

Renal cell carcinoma (RCC) is one of the most common cancers, accounting for 5% and 3% of all new malignancies in men and women in the United States, respectively [1]. It is estimated that in 2025, 52,410 new cases of RCC will occur in men and 28,570 in women, highlighting the current relevance of this disease [1].

Chromophobe renal cell carcinoma (chRCC) is a subtype that accounts for around 5% of all RCC and is the most common non-clear cell RCC in young women [2].

Due to its histologic similarities to renal oncocytoma (RO), differentiating chRCC from RO can be challenging. Therefore, this review discusses the radiological, macroscopic, microscopic and immunohistochemical features that distinguish these entities.

In addition, many new insights into the genome, epigenome, transcriptome and proteome of chRCC have been gained in recent years, which could contribute to a better understanding of this tumor and facilitate its differentiation from similar neoplasms. Furthermore, the course of the disease could be predicted more reliably and, in the rare case of metastases, stratified therapeutic approaches could be used.

## Clinical appearance and etiology

ChRCC often occurs sporadically and is often discovered as an incidental finding at early tumor stage. The incidence peaks around the age of 60, but a higher incidence of chRCC is also observed in young women, suggesting a correlation with hormonal balance [2]. Most patients have no symptoms at the time of diagnosis, but complaints such as flank pain, macrohematuria, pyelonephritis and a palpable upper abdominal tumor can also occur.

ChRCC is usually clinically characterized by less malignant and aggressive behavior and rarely shows signs of dedifferentiation [3]. Metastases therefore only occur in very few patients, primarily hematogenously to the liver or lungs [4]. The prognosis of chRCC is therefore significantly better than that of clear cell or papillary RCC, which will be discussed in more detail below.

The etiology of chRCC is still unclear, but smoking, obesity, arterial hypertension and familial predisposition appear to be important risk factors.

In addition, chRCC occurs more frequently in connection with Birt-Hogg-Dubé syndrome (BHDS), which is caused by a mutation in the *FLCN* gene. BHDS is an autosomal dominant hereditary disease that can be characterized by fibrofolliculomas, pulmonary and renal cysts and a nearly 50-fold increased risk of spontaneous pneumothorax. Patients with BHDS also have an increased risk of developing renal oncocytomas, papillary RCC or hybrid oncocytic/ chromophobe tumors (HOCT) [5]. These HOCT associated with BHDS tend to present more frequently as multifocal lesions compared to chRCC and RO, which may serve as an initial clinical indicator of this tumor subtype [5].

In addition, Cowden syndrome, which is primarily caused by *PTEN* mutations, and tuberous sclerosis, which is often associated with *TSC1/2* mutations, are both associated with an increased risk of developing chRCC [6].

Overall, many chRCC cases occur sporadically and are characterized by an indolent course of the disease, which is also reflected in the pathological features of these neoplasms.

## Radiological features

Although no pathognomonic features for chRCC exist on cross-sectional imaging, both CT and MRI scans offer valuable diagnostic insights [7]. Specifically, chRCC typically presents as a solid tumor mass on both CT and MRI, which is usually well-demarcated from surrounding tissues [7]. On CT, calcification is observed in 14–34% of cases, and a central scar is seen in 19–34% of cases [7]. Furthermore, chRCC demonstrates moderate contrast enhancement, typically most prominent during the nephrographic phase, or, in rarer cases, in the corticomedullary phase; however, the enhancement is lower than that of the renal cortex in all phases [7]. Additionally, the enhancement in chRCC is generally less than that of ccRCC [7].

On MRI, chRCC is identified as a solid tumor mass, which appears uniformly hypointense on T1-weighted images and tends to show a heterogeneous appearance on T2-weighted images [7]. Similar to CT, MRI enhancement in chRCC is consistently lower than that of the renal cortex throughout all phases [7]. Moreover, necrosis or cystic changes are rare in chRCC [7].

## Pathology

ChRCC is histopathologically characterized by shrunken cell nuclei with perinuclear halos, which is why it can usually be easily distinguished from clear cell renal cell carcinomas. However, chRCC, like benign RO, is derived from cells of the collecting ducts of the kidney, resulting in morphological similarities, which can present pathologists with diagnostic challenges [6]. The characteristic features of chRCC in terms of macroscopy, microscopy and immunohistochemistry are discussed below.

### Macroscopic features

Macroscopically, chRCC is characterized by a light brown to grayish colored cross-section and the presence of a pseudocapsule, which visibly separates the tumor from the surrounding tissue [8]. The cut surface of chRCC is often homogeneous, and necrosis and central scarring rarely occur. Moreover, chRCC usually remains confined to the renal parenchyma, without infiltration of the perirenal adipose tissue, lymph nodes, blood vessels or perineural sheaths, which may partly contribute to its favorable prognosis.

Benign RO is also clearly distinguishable from the surrounding tissue; however, it typically lacks a capsule, and central scarring is frequently observed. The cut surface is usually homogeneous and mahogany-colored [9].

### Microscopic features

Microscopically, the classic type of chRCC must be distinguished from its eosinophilic subvariant, although both subtypes share characteristic features such as strongly shriveled, irregularly angled, hyperchromatic ("raisinoid") nuclei and perinuclear brightening (halos) [10]. Binuclear cells and well-defined cell borders are also characteristic features. Tumor cells generally appear atypical, and signs of dedifferentiation are rare. In such cases, dedifferentiation can be classified as sarcomatoid, glandular and anaplastic [11]. While anaplastic dedifferentiation is characterized by large epithelioid cells with hyperchromatic, pleomorphic nuclei and highly prominent nucleoli, glandular dedifferentiation consists of cuboidal cells arranged in tubules and micropapillae [11]. Several forms of dedifferentiation can also occur in one histological preparation [11].

The classic type is primarily composed of pale, polygonal cells with raisinoid nuclei and "plant cell-like" cell membranes, which may exhibit trabecular growth patterns. In addition to these pale cells, eosinophilic cells are also occasionally found, but only in small numbers.

The eosinophilic variant, by contrast, contains a high proportion of eosinophilic cells with granular cytoplasm and numerous mitochondria, while pale cells occur only occasionally.

This eosinophilic subtype is therefore often difficult to distinguish morphologically from RO, as both exhibit highly eosinophilic cytoplasm, primarily due to their abundant mitochondria. In addition, RO consists of round and vesicular cells with prominent nucleoli, which also occur more frequently in chRCC. Occasionally, RO is also characterized by mitoses, necrosis and binuclear cells, but no raisinoid nuclei with perinuclear halos, which remains a key diagnostic feature of chRCC.

In this context, HOCT are characterized by the coexistence of these chromophobe-like and oncocytoma-like cells within the same tumor. These cells exhibit diverse growth patterns and may display eosinophilic or clear cytoplasm along with round nuclei and perinuclear halos [5].

**Fig. 1:**
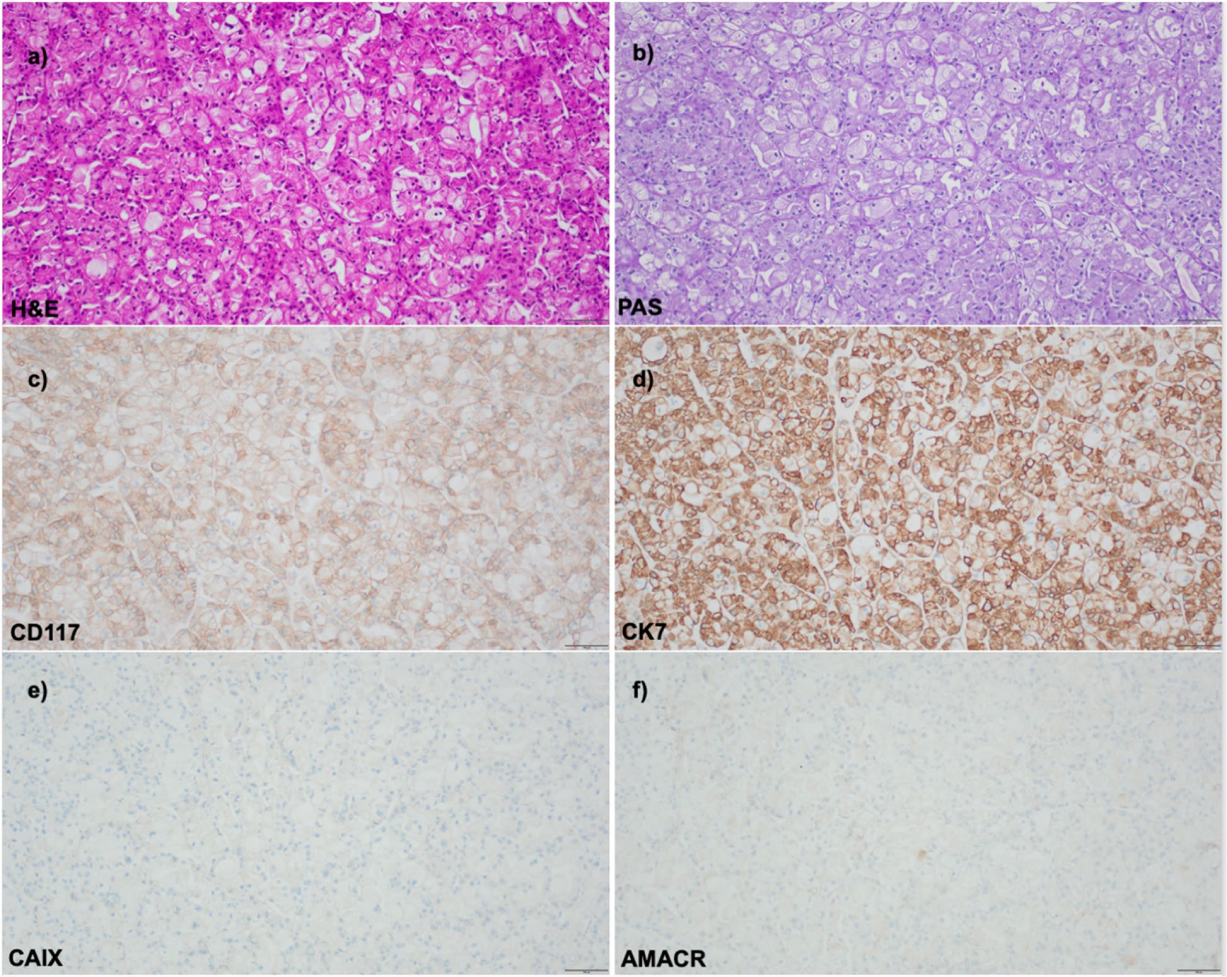
**a-f** Typical histology of chromophobe renal cell carcinoma revealing partly eosinophilic cytoplasm (**a**), partly prominent cytoplasmic membranes without PAS-positive glycogen (**b**). Immunohistochemistry shows positive staining for CD117 (**c**) and CK7 (**d**) and negativity for CAIX (**e**) and AMACR (**f**). Bars represent 100 µm

Although these morphological characteristics provide valuable diagnostic clues for differentiating chRCC from RO and HOCT, their recognition can be challenging in routine practice [9]. In such cases, immunohistochemical markers must be employed to establish a definitive diagnosis, as discussed in the following section. Fig. 1.

### Immunohistochemistry

The immunohistochemical detection of antigens using antibodies and visualization systems often enables the targeted in situ analysis of specific protein expression or modifications. This approach allows conclusions to be drawn regarding the tissue type or tumor under investigation. In the case of chRCC, immunohistochemical methods are used to ensure clearer differentiation from RO and ccRCC.

Fundamentally, ccRCC is negative for CK7, EpCAM, claudin 7 and for Hale staining, while it is positive for vimentin, CAIX and GST-alpha detection [9, 12]) (see Table 1).

In contrast, both chRCC and RO do not exhibit immunopositivity for vimentin, CAIX or GST-alpha [12]. However, CkAE1/AE3, LMWCK (CAM5.2) and Ksp-cadherin are immunopositive in both chRCC and RO [12] (see Table 1).

The EpCAM and CK7 antigens are only detectable in chRCC, whereas they are not immunopositive in RO (see Table 1). Hale staining and claudin 7 are also positively detected in chRCC, while they only appear focally positive or even negative in RO [9] (see Table 1).

Another important marker is C-Kit (CD117), which is immunopositive in both chRCC and RO, but immunonegative in ccRCC. C-Kit is a receptor located on the cell membrane of various somatic cells, belonging to type 3 of the receptor tyrosine kinase family. It plays key roles hematopoiesis, angiogenesis, melanogenesis and gametogenesis. Mutations in the corresponding coding gene *C-kit* can lead to functional dysregulation and thus contribute to the development of tumors. In 2023, Jiang et al. reported that the expression of the protooncogene *c-kit* is significantly higher in tissues of chRCC from smokers than in those from nonsmokers, indicating the relevance of smoking for the development of molecular aberrations in chRCC [13]. In this context, HOCT likewise typically demonstrate immunopositivity for C-Kit and CK7, while lacking expression of CAIX [5].

Furthermore, the immune marker phospho-S6 can be used to quantify the potential molecular activity of mTORC1. It exhibits significantly increased immunopositivity in dedifferentiated tumor tissue and can be used as an immunohistochemical marker for dedifferentiation if certain tumor areas cannot be clearly characterized [11].

Additionally, immunohistochemical detection of progesterone receptors can be applied, as they are predominantly positive in both chRCC and RO, while they remain negative for other renal cell carcinomas with oncocytic cytoplasm [14]. This frequently observed increased expression of progesterone receptors in chRCC and in RO could indicate an enhanced progesterone sensitivity of these two tumors. Therefore, it is an interesting question whether the use of hormonal contraceptives, similar to breast cancer, might be contraindicated, as this could promote the growth of chRCC and RO. This hypothesis is supported by statistical observations by Daugherty et al., which suggest that chRCC occurs more frequently in young women. Since high progesterone levels are also present during pregnancy, this could further contribute to the growth of chRCC and RO. (see Table 1).

**Table 1:** Immunohistochemical differentiation between chRCC, RO, and ccRCC

	**chRCC**	**RO**	**ccRCC**
C-Kit	+ (diffuse)	+ (diffuse)	-
CK7	+ (> 70%, diffuse)	± (< 10%, in individual cells)	-
EpCAM	+	-	-
Hale staining	+	+ (luminal)	-
Claudin 7	+ (> 70%, membrane)	- (< 10%, in individual cells)	-
Vimentin	-	-	+
GST-alpha	-	-	+
CkAE1/AE3	+	+	+
LMWCK (CAM5.2)	+	+	+
Ksp-cadherin	+	+	n/a
CAIX	-	-	+
Progesterone receptor	+	+	n/a

*ChRCC* chromophobe renal cell carcinoma, *RO* renal oncocytoma, *ccRCC* clear cell renal cell carcinoma, *n/a* not available

Source: based on Lüders et al., 2016 and [15]

## Molecular biology

Recent molecular biological studies on chRCC have provided profound insights into the development and evolution of these tumors. The following section will therefore discuss findings from genome, epigenome, transcriptome and proteome analyses concerning the unique features of chRCC and their potential new clinical significance.

### Genomic alterations in chRCC

The genome of sporadically occurring chRCC often shows aneuploidy. Most of the non-dedifferentiated chRCC presented losses of chromosomes of type 1, 2, 6, 10, 13 and 17. Changes in the karyotypes of chromosomes 3, 5, 8, 9, 11, 18 and/or 21 are observed in 12% to 58% of cases [16]. These aneuploidies are observed significantly more frequently in the histologically classic subtype of chRCC than in the eosinophilic subtype, indicating further genomic differences between these two variants of chRCC [16].

Furthermore, it is assumed that additional genomic alterations occur in chRCC cells with increasing degrees of dedifferentiation [11]. While well-differentiated cells of classical chRCC are characterized by hypodiploidy, dedifferentiated cells usually exhibit a completely diploid or even hyperdiploid chromosome set with two or more copies of chromosomes 1, 2, 6, 10, 13, 17 and 21 [11].

This finding suggests that the dedifferentiated tumor cells develop from the classic tumor cells of chRCC. In this regard, it appears that the monosomes present in the histologically classic tumor cells are duplicated through amplification to generate the dedifferentiated tumor cells [11].

In addition, the analysis of genetic changes shows that the *TP53* gene is frequently mutated in chRCC, leading to a loss of function of p53 as a consequence [16]. These mutations can be broadly interpreted as reduced protection of the genome by the "guardian of the genome", *TP53*.

Notably, if a *TP53* mutation is found in classical tumor cells within a tumor, the same mutation is also observed in the dedifferentiated tumor cells [11]. This *TP53* mutation is hemizygous in classical tumor cells and homozygous in the corresponding dedifferentiated tumor cells [11]. A similar observation can be made for the *PTEN* mutation in chRCC on chromosome 10. It is therefore assumed that *TP53* and *PTEN* mutations precede the aforementioned amplification and arise before the development of dedifferentiation in tumor cells [11].

In addition, mutations in the *MTOR, NRAS, TSC1* and *TSC2* genes have previously been described [16]. However, according to the 2022 WHO classification, renal tumors with alterations in the mTOR pathway, particularly biallelic loss of TSC1 or TSC2, are now classified as eosinophilic solid and cystic renal cell carcinoma (ESC-RCC), a distinct entity characterized by mTORC1 pathway activation rather than being considered a subtype of chRCC [17].

Alongside aneuploidies, increased genomic rearrangements to recurrent structural breakpoints in the *TERT* promoter region have been found in chRCC. These effects are functionally associated with increased *TERT* expression and, as a result, increased transcriptional activity of the promoter [16]. Since *TERT* has important functions in telomere maintenance and DNA repair, it is assumed that these *TERT*-associated rearrangements and dysregulations could contribute to tumorigenesis [16].

Another important aspect is mutations in the genes of the *HER* family ("human epidermal growth factor receptors"), which consists of *HER1, HER2, HER3* and *HER4*. Weng et al. have shown that loss of expression of the *HER2* gene is frequently observed in chRCC. This could be explained by the localization of the *HER2* gene on chromosome 17q12 and the frequently observed chromosomal deletion of this chromosomal segment [18]. However, loss of expression of *HER2* could not be statistically associated with tumor progression or aggressiveness [18].

Furthermore, chromosomal rearrangements in the *HER1* and *HER3* genes have been observed in chRCC, which were attributed to the insertion of an unknown gene [18]. As a result, altered gene functions and abnormal transduction activities with possible effects on tumor induction and tumor progression are discussed [18].

Additionally, alterations in the mitochondrial DNA of chRCC have been detected, with the *MT-ND5* gene being affected [16]. This gene is important for the functionality of complex 1 of the respiratory chain [16]. Complex 1 of the respiratory chain was altered in 18% of the cases reported by Davis et al. Table 2.

**Table 2:** List of chRCC associated gene mutations

*Gene*	Location	Gene Type	Type of Mutation
*TP53*	17p13.1	Tumor suppressor gene	Heterozygous copy loss, somatic mutation (32%)
*PTEN*	10q23.31	Tumor suppressor gene	Heterozygous copy loss, non-silent mutation
*MTOR*	1p36.22	Oncogene	Heterozygous copy loss, somatic mutation (3%)
*NRAS*	1p13.2	Oncogene	Heterozygous copy loss, somatic mutation (1,5%)
*TSC1*	9q34.13	Tumor suppressor gene	Heterozygous copy loss, somatic mutation (3%)
*TSC2*	16p13.3	Tumor suppressor gene	Heterozygous copy loss, somatic mutation (3%)
*HER1 (EGFR)*	7p11.2	Oncogene	Chromosomal rearrangements due to insertion of an undefined gene
*HER2 (ERBB2)*	17q12	Oncogene	Deletion
*HER3*	12q13.2	Oncogene	Chromosomal rearrangements due to insertion of an undefined gene
*MT-ND5*	Mitochondrial DNA	Mitochondrial gene	High heteroplasmy

Source: Based on [11, 16, 18]

### Epigenome

The epigenome encompasses a wide range of mechanisms that regulate gene expression independently of changes in the DNA sequence. In this regard, epigenetic modifications and changes in interactions between epigenome and genome may be significantly involved in the process of tumorigenesis [19]. According to the stem cell hypothesis, these epigenetic changes occur in somatic cancer stem cells and can be passed on to daughter cells, promoting carcinogenesis and tumor growth [20]. Such changes in the epigenome have been described for chRCC, where they may contribute to tumor development and progression, as discussed in the following section.

Epigenetic modifications involving DNA methylation and demethylation play important roles in carcinogenesis [19]. Tumor cells have been shown to exhibit both DNA hypermethylation and simultaneous global hypomethylation in different genomic regions [19]. These DNA hypomethylations occur earlier and are more strongly associated with chromosomal instability [21]. Hypermethylation occurs through DNA methyltransferases in CpG islands, which are more frequently found in the promoter regions of tumor suppressor genes, leading to the inactivation of these genes [19, 21]. Overall, global hypomethylation results in genetic instabilities, while hypermethylation leads to the inactivation of tumor suppressor genes [19]. These processes have a significant influence on carcinogenesis.

In this context, Faraj Tabrizi et al. reported that increased DNA methylation of the *cadherin3* gene (*CDH3*) may be associated with higher tumor stages and higher degrees of differentiation in clear cell RCC [22]. Future research could explore changes in the expression of *CDH3* and DNA methylation of *CDH3* in chRCC to draw conclusions about potential correlations with the extensive atypia of chRCC and their tumor behavior.

Another important component of the epigenome is microRNAs (miRNAs), which are non-coding RNA molecules approximately 19–25 nucleotides long [23]. MiRNAs play a important role in post-transcriptional gene regulation by binding to messenger RNA (mRNA), thereby influencing gene expression [23]. In this context, certain genes coding for miRNAs may be overexpressed, causing them to act as oncogenes that promote tumor progression. Conversly, downregulation of tumor-suppressive miRNAs can promote carcinogenesis. Thus, we hypothesize that the loss of chromosomes 1, 2, 6, 10, 13 and 17 may also lead to the loss of genes encoding miRNAs with tumor suppressive functions. This could result in downregulation of such miRNAs, thereby promoting tumor progression in chRCC. Given the limited research on miRNAs in chRCC, further studies are necessary to improve the understanding of these molecules and to develop potential miRNA-based therapeutic approaches for the treatment of chRCC.

Another important component of the epigenome is long noncoding RNAs (lncRNAs), which consist of more than 200 nucleotides and can also regulate gene expression [24]. Tumor-specific lncRNAs in chRCC have been significantly associated with tumor progression and tumor-specific survival [24]. High expression of the lncRNAs COL18A1-AS1, BRE-AS1, SNHG7, TMEM51-AS1, C21orf62-AS1 and LINC00336, along with low expression of LINC00882, are significantly positively associated with tumor-specific overall survival [24] (see Table 3).

**Table 3:** List of lncRNAs in chRCC significantly associated with cancer-specific overall survival

lncRNA	Association with Prolonged Overall Survival	*P*-Values
COL18A1-AS1	High expression	*P* = 0.009
BRE-AS1	High expression	*P* = 0.011
SNHG7	High expression	*P* = 0.014
TMEM51-AS1	High expression	*P* = 0.024
C21orf62-AS1	High expression	*P* = 0.027
LINC00336	High expression	*P* = 0.037
LINC00882	Low expression	*P* = 0.047

Source: based on [24]

According to the "competitive endogenous RNA" (ceRNA) hypothesis, lncRNAs, pseudogene RNAs and mRNAs communicate with each other by binding to the same miRNA through so-called "miRNA response elements" (MREs), thereby competing for the miRNA [24, 25]. The main idea of this hypothesis is that miRNAs are blocked by the MRE-mediated binding of lncRNAs and pseudogene RNAs. As a result, these miRNAs can no longer bind to mRNAs, leading to a shift in the regulation of gene expression.

Overall, this demonstrates that characteristic lncRNAs, miRNAs and pseudogene RNAs play a central role in tumorigenesis and in the tumor biology of chRCC. They should, therefore, be considered as key research targets for therapeutic and diagnostic strategies in the future.

### Transcriptome

The transcriptome comprises the entire spectrum of genes transcribed into RNA within a cell at a given time, allowing conclusions about the activity and gene expression profiles of tumors such as chRCC. In addition to immunohistochemical methods, transcriptomic differences can be analyzed to facilitate the differentiation between eosinophilic-type chRCC and RO for clinical diagnosis.

To this end, Satter and Tran et al. have developed a signature model consisting of 30 genes that exhibit significantly different expression levels in chRCC and RO. This model can distinguish the extensive transcriptomic differences between the two tumors with an accuracy of 97.8% [26].

This model, termed COGS (chromophobe and oncocytoma related gene signature), includes the *AP1M2* gene, which encodes a subunit of the clathrin-associated adapter protein complex 1 and thus assumes important functions in protein sorting in the trans-Golgi network and in endosomes [27]. These findings indicate that both tumor types exhibit fundamentally different properties and follow different pathways of protein sorting.

Additionally, *PLCL1* and *PLCL2* show differential expression in chRCC and RO. *PLCL1* encodes proteins involved in phospholipid-based intracellular signaling cascades, whereas *PLCL2* is thought to regulate Ins(1,4,5)P53 in the endoplasmic reticulum [27]. Another gene within the COGS is *ITGB3*, which encodes a protein of the FTS/ Hook/ FHIP complex and may thereby facilitate vesicle transport via the homotypic vesicular protein sorting complex [27]. These expression alterations in *PLCL1, PLCL2* and *ITGB3* could could potentially influence tumor development and tumor progression.

A further important gene for differentiating these tumor types is the *BSPRY* gene, which regulates epithelial calcium transport by inhibiting TRPV5 activity [27]. Altered *BSPRY* expression could thus contribute to the reduced TRPV5 expression observed in renal cell carcinomas and may be associated with the altered expression of the vitamin D receptor [28]. Disruptions in calcium homeostasis and impaired vitamin D signaling could therefore be significantly involved in tumor development and tumor progression in chRCC.

This gene signature model holds promise as a complementary tool to immunohistochemistry for the clinical diagnosis of chRCC and offers a high degree of diagnostic certainty. However, further validation studies are still required.

### Proteome

In addition to the transcriptome, the proteome can also provide insights into tumor biology. Proteomics encompasses the entirety of proteins present in a cell and is used to identify the characteristic properties of certain tissues and tumors.

Both chRCC and RO exhibit alterations in protein amino acid, lipid and carbohydrate metabolism compared to normal adjacent tissue [29]. Furthermore, dysregulations in mitochondrial metabolism have been observed [29]. The proteomes of chRCC and RO share greater similarities with each other than with normal adjacent tissue, which aligns with their phenotypic and histologic resemblance [29].

This is particularly evident in the fact that proteins involved in gluconeogenesis, such as fructose-1,6-bisphosphatase and pyruvate carboxylase, and proteins involved in fatty acid synthesis, such as alcohol dehydrogenase 1b, are significantly downregulated in both tumors compared to normal adjacent tissue [29, 30]. Additionally, amino acid metabolism is significantly downregulated in both tumors, as demonstrated by the reduced expression of argininosuccinate synthetase, phosphoglycerate dehydrogenase and glutamate oxaloacetate transaminase 1 and 2 [29]. This downregulation of amino acid metabolism, and the associated deficiency of amino acids such as arginine or glycine supports the hypothesis of amino acid auxotrophy in these tumors [29, 31]. Consequently, tumor cells must obtain these essential amino acids from the normal surrounding tissue [29, 31]. It is plausible that normal adjacent tissue compensates for these metabolic alterations by upregulating the amino acid metabolism.

Nevertheless, chRCC also exhibits important unique characteristics. Firstly, proteins involved in oxidative phosphorylation pathway are less dysregulated in chRCC compared to RO [29]. Additionally, proteins from other mitochondrial metabolic pathways, including those of the respiratory chain, show significantly greater dysregulateion in RO than in chRCC [29].

These findings indicate distinct mitochondrial metabolic pathways and substantial mutations in mitochondrial DNA [29].

A significantly higher number of mutations in mitochondrial genes in RO results in a large proportion non-functional mitochondria. Their function may be partially compensated by the import of functional mitochondria from the normal surrounding tissue into the respective tumor cells [32–34]. However, these mutations lead to an accumulation of defective mitochondria within the cells, suggesting a correlation between chronic metabolic disorder and impaired autophagy [32].

These mitochondrial defects and disruptions in cellular respiration contribute to chronic metabolic disorders in RO, leading to the activation of AMP kinase and the downregulation of mTOR [32]. Activation of AMP kinase during transient energy crises normally leads to the degradation of the Golgi apparatus in order to inhibit trafficking and conserve energy [32]. This Golgi apparatus is subsequently rebuilt, allowing the cells to resume growth. However, in RO cells, persistent activation of AMP kinase results in the irreversible disassembly of the Golgi apparatus, leading to impaired autophagy due to ongoing metabolic dysfunction and chronic energy shortages [32]. Inhibition of complex 1 of the respiratory chain plays a crucial role in this process. Ultimately, these mechanisms contribute to the slower growth rate of RO cells [32].

In contrast, *PTEN* and *P53* mutations in chRCC attenuate AMP kinase activation, thereby preserving Golgi function and autophagy [32]. These findings suggest a comparatively faster growth rate of chRCC.

## Therapeutic approaches

While monitoring of the tumor is sufficient for RO, with nephron-sparing removal only necessary if the tumor increases in size, both partial and radical nephrectomies are the gold standard of curative therapy for chRCC in cases where resection is feasible.

Although chRCC generally has a better prognosis than ccRCC, metastatic chRCC is associated with a worse prognosis than recurrent ccRCC [35]. One reason for this is the rarity of these cases, leading to a lack of meaningful studies and limited therapeutic successes in the treatment of metastatic chRCC.

As metastatic chRCC is highly resistant to chemotherapeutic agents, treatments with various immunotherapies are currently preferred. In this context, the phase 2 study SUNNIFORECAST is the first to investigate the therapeutic effects of the combination therapy with nivolumab and ipilimumab compaired to the monotherapy sunitinib as first-line therapy in patients with unresectable or metastatic non-clear cell renal cell carcinoma in a randomized, open comparative study [36]. Results from phase 1 studies have already provided indications that the combination therapy of nivolumab and ipilimumab could achieve promising results, which is now being evaluated in the SUNNIFORECAST study [36]. The primary endpoint of this study is survival (OS) at 12 months, while survival rates at 6 and 12 months, progression-free survival (PFS), median overall survival (mOS), objective response rate (ORR) and duration of response (DOR) are secondary endpoints of this study [36]. Further exploratory objectives include evaluatiing the safety, tolerability and immunogenicity of this combination therapy [36]. Overall, the SUNNIFORECAST trial has great potential to provide groundbreaking findings for the first-line treatment of unresectable or metastatic non-clear cell, particularly chromophobe, renal cell carcinoma and to act as an important guide for treating physicians.

Furthermore, Zhang et al. report a case in which combination therapy with sintilimab, axitinib and denosumab achieved promising results in a patient with distant metastases of chRCC in the lungs and bones. Sintilimab is a recombinant human monoclonal IgG4 anti-PD-1 antibody that binds to PD-1 receptors and thus blocks the binding between PD-1 receptors and its ligands. This restores the endogenous anti-tumor T-cell response [35]. Axitinib, on the other hand, is a highly selective tyrosine kinase inhibitor of vascular endothelial growth factor receptors with high target affinity and strong specificity [35]. Finally, denosumab is a monoclonal antibody of the RANKL inhibitors and was administered to treat bone metastases. The combination of sintilimab immunotherapy and axitinib-based targeted therapy led to a significant clinical improvement in the patient and may serve as a potential therapeutic approach for clinicians.

However, as the general efficacy of these therapies has not yet been adequately demonstrated and axitinib can cause serious side effects such as high blood pressure, fatigue and body aches, further studies on this treatment are required. In this regard, various checkpoint inhibitors could be considered in clinical practice for patients with dedifferentiation or distant metastases to achieve potential therapeutic benefits.

## Grading and prognosis of chromophobe renal cell carcinoma

The World Health Organization/ International Society of Urologic Pathologists (WHO/ ISUP) grading system is currently the most widely used system for assessing the aggressiveness of RCC [37]. However, this grading system is of limited prognostic value, particularly for chRCC, as these tumors are inherently characterized by core atypia. Consequently, a grading system is not applied in chRCC [38]. Therefore, it is crucial to establish an appropriate grading system for chRCC in the future that can reliably classify the aggressiveness and prognosis of these tumors.

In 2010, Paner et al. developed a three-stage grading system for chRCC, distinguishing grades 1 and 2 based on nuclear crowding and diffuse atypia of the tumor cells, with necrosis playing only a minor role in both grades [39]. In contrast, grade 3 is characterized by sarcomatoid dedifferentiation and frank anaplasia [39, 40].

Building on this, Avulova et al. proposed a four-level grading system for chRCC an extension of Paner et al.’s classification. The four levels of the grading system are defined as follows:

ChRCC is classified as grade 1 if there is wide nuclear spacing, no nuclear crowding or diffuse atypia [40]. In grade 1, it is not significantly important under these circumstances whether coagulation necrosis is present or not. Grade 2 is characterized by nuclear compaction (high nuclear/cytoplasmic density and contacts between the nuclei) and the presence of atypical cells (at least triple nuclear size differences with irregular chromatin) without coagulation necrosis [15]. Grade 3 is achieved when the conditions of grade 2 are met and coagulation necrosis is also present, and grade 4 is achieved when sarcomatoid dedifferentiation or anaplasia is detectable in the tumor cells [40].

Liu et al. applied this four-stage classification system to a cohort of 263 patients with chRCC and obtained the following prognostic predictions: The cancer-specific survival (CSS) for 5 and 10 years were 95.9% and 94.2% respectively [15]. When stratified by Avulova et al.’s grading system, the CSS at 5 years were 100.0%; 91.4%; 82.1% and 37.5% for grades 1, 2, 3 and 4, respectively, while the 10-year CSS rates were 98.8%; 91.4%; 82.1% and 0% [15] (see Table 4). These results underscore the generally favorable prognosis of chRCC.

The classification system according to Avulova et al. is therefore significantly associated with CSS and the occurrence of metastases and has significantly better prognostic ability than the Fuhrman grading system [15].

**Table 4:** Four-tier Classification System for chRCC by Avulova et al. with Cancer-Specific Survival (CSS) for 5 and 10 Years According to Liu et al

*Grade*	Characteristics	5-Year CSS	10-Yeat CSS
*1*	Wide nuclear spacing, no nuclear crowding or diffuse atypia present, with or without coagulative necrosis	100,0%	98,8%
*2*	Nuclear crowding with high nucleus/cytoplasm density, as well as contacts between nuclei, and the presence of atypical cells (at least threefold differences in nuclear size with irregular chromatin) without coagulative necrosis	91,4%	91,4%
*3*	Nuclear crowding (high nucleus/cytoplasm density, as well as contacts between nuclei) and the presence of atypical cells (at least threefold differences in nuclear size with irregular chromatin) with coagulative necrosis	82,1%	82,1%
*4*	Sarcomatoid dedifferentiation or anaplasia	37,5%	0,0%

*CSS* Cancer-specific survival

However, a notable limitation of the prognostic utility of Avulova et al.’s classification system is that, while the distinction between grades 2 and 3 provides insights about the aggressiveness of the tumor, no significant differences in prognosis have been observed between these two grades [15].

Overall, both the grading systems proposed by Paner et al. and Avulova et al. are significantly associated with CSS and metastasis risk. However, there is disagreement about their better prognostic ability.

Moreover, age, TNM status, tumor size, necrosis, dedifferentiation, lymphovascular invasion and the classification according to the four-stage grading system have been shown to significantly correlate with the aggressiveness of chRCC [15, 40, 41].

## Conclusion

In this review, we have outlined the key pathologic and molecular biological characteristics of chRCC and provided clinical decision support for treating physicians.

In addition to the microscopic features of chRCC, such as the raisinoid cell nuclei with perinuclear halos, immunohistochemical markers should be utilized to more clearly differentiate chRCC from RO. Beyond the routinely used markers like C-Kit and CK7, progesterone receptors should also be immunohistochemically stained to assess the individual progesterone sensitivity of chRCC. As previously described, many chRCC exhibit increased levels of progesterone receptors, suggesting notable progesterone sensitivity [14]. Therefore, we propose that chRCC can be considered as a contraindication for the use of hormonal contraceptives, similar to breast cancer, to avoid faster tumor growth. Additionally, phospho-S6 can indicate mTORC1 activity and serve as an immunohistochemical marker for more precise identification of dedifferentiated tumor tissue [11].

ChRCC also exhibit distinctive molecular biological characteristics, enabling their precise differentiation from RO and other renal neoplasms. For example, the genome of chRCC is typically characterized by an aneuploid chromosome set in classic tumor cells, while dedifferentiated tumor cells are either diploid or hyperdiploid [11]. Moreover, *TP53* and *PTEN* mutations occur more frequently, indicating the association of these genes with the tumor biology of chRCC [16]. In terms of the epigenome, lncRNAs and miRNAs may play a crucial role in carcinogenesis and tumor progression in chRCC, warranting further investigation. Given the relevance of *CDH3* gene methylation in clear cell RCC, additional studies on its impact on chRCC are needed. At the transcriptomic level, a signature model (COGS) presented by Satter and Tran et al. effectively distinguishes chRCC and RO based on their transcriptomic properties. The COGS model includes genes such as *AP1M2, PLCL1, PLCL2, ITGB3* and *BSPRY*, whose altered expression could contribute to tumorigenesis and tumor progression.

These genetic, epigenetic, transcriptomic, and proteomic alterations, including the COGS, are primarily applied in experimental settings due to their recent discovery and relevance. In the future, however, they should be increasingly integrated into clinical routine practice, particularly when the differential diagnosis between chRCC and RO by immunohistochemistry is not sufficient.

With regard to the treatment of metastatic chRCC, immunotherapies have demonstrated effectiveness in certain cases [35]. We believe that the innovative approach of the SUNNIFORECAST study holds great potential to provide important insights into the first-line treatment of unresectable or metastatic chRCC, thereby serving as a model for future clinical treatment decisions.

Furthermore, we consider that the use of checkpoint inhibitors represents a promising approach for the treatment of dedifferentiated forms of chRCC. However, further meaningful clinical studies are needed to better investigate the effect of individual checkpoint inhibitors. The small number of metastatic and dedifferentiated chRCC may represent a limitation in this context.

Overall, chRCC typically follow an indolent disease course and present challenges in classification due to the extensive atypia, pleomorphic cell nuclei and often prominent nucleoli [11]. Nevertheless, it is evident that factors such as age, TNM status, tumor size, necrosis, dedifferentiation, lymphovascular invasion, and the classification within the four-stage grading system proposed by Avulova et al. have strong prognostic value. Moving forward, these parameters should be integrated into the development of a universally accepted classification system, which should also be included in clinical guidelines to allow for uniform grading and prognostication of chRCC.

## Data Availability

No datasets were generated or analysed during the current study.

## Abbreviations

BHDS: Birt-Hogg-Dubé Syndrome
ccRCC: Clear cell Renal Cell Carcinoma
ceRNA: Competitive endogenous RNA
chRCC: Chromophobe Renal Cell Carcinoma
CSS: Cancer-Specific Survival
DOR: Duration of Response
ESC-RCC: Eosinophilic Solid and Cystic Renal Cell Carcinoma
HOCT: Hybrid Oncocytic/Chromophobe Tumor
ICI: Checkpoint Inhibitor
lncRNA: Long noncoding RNA
miRNA: MicroRNA
mOS: Median Overall Survival
MRE: MiRNA Response Element
mRNA: Messenger RNA
ORR: Objective Response Rate
OS: Overall Survival
PFS: Progression-Free Survival
RCC: Renal Cell Carcinoma
RO: Renal Oncocytoma
